# Overexpression of inhibitor of DNA-binding (ID)-1 protein related to angiogenesis in tumor advancement of ovarian cancers

**DOI:** 10.1186/1471-2407-9-430

**Published:** 2009-12-10

**Authors:** Min Khine Maw, Jiro Fujimoto, Teruhiko Tamaya

**Affiliations:** 1 Department of Obstetrics and Gynecology, Graduate School of Medicine, Gifu University School of Medicine, 1-1 Yanagido, Gifu City 501-1194, Japan

## Abstract

**Background:**

The inhibitor of DNA-binding (ID) has been involved in cell cycle regulation, apoptosis and angiogenesis. This prompted us to study ID functions in tumor advancement of ovarian cancers.

**Methods:**

Sixty patients underwent surgery for ovarian cancers. In ovarian cancers, the levels of ID-1, ID-2 and ID-3 mRNAs were determined by real-time reverse transcription-polymerase chain reaction. The histoscore with the localization of ID-1 was determined by immunohistochemistry. Patient prognosis was analyzed with a 36-month survival rate. Microvessel counts were determined by immunohistochemistry for CD34 and factor VIII-related antigen.

**Results:**

ID-1 histoscores and mRNA levels both significantly (p < 0.001) increased in ovarian cancers according to clinical stage, regardless of histopathological type. Furthermore, 30 patients with high ID-1 expression had a lower survival rate (53%) compared to patients with low ID-1 expression (80%). ID-1 histoscores and mRNA levels significantly (p < 0.0001) correlated with microvessel counts in ovarian cancers.

**Conclusion:**

ID-1 increased in ovarian cancer cells during tumor progression. Moreover, ID-1 expression levels correlated with microvessel counts. Therefore, ID-1 might work on tumor advancement via angiogenesis and is considered to be a candidate for a prognostic indicator in ovarian cancers.

## Background

Inhibitor of DNA binding (ID) proteins are members of a family of basic helix-loop-helix (bHLH) transcription factors lacking the DNA-binding domain [1]. ID acts as dominant-negative regulators of bHLH proteins by forming transcriptionally inactive Id-bHLH protein complexes [2,3]. ID has been implicated in different steps in tumorigenesis, differentiation and metastasis [4-9].

ID-1 induces cell proliferation, increases DNA synthesis, and immortalizes mammalian cells in corporation with some oncogenes [10,11]. Overexpression of ID-1 inhibits expression of p16 [12,13], p21 [14] and p27 [15], which leads to increased activity of cyclin dependent kinase 2 (CDK2) and increased phosphorylation of retinoblastoma protein. Therefore, the increased liberation of ID-2 from retinoblastoma protein and more free-ID-2 is available for the inhibition of E proteins to facilitate proliferation [16]. ID-1 interacts with various cell cycle regulators [12,17] and causes cells to pass a mitogen-restricted point in late G1 phase [18]. Therefore, ID-1 is responsible for some changes in gene expression that lead to growth and invasion of tumor cells [19]. Moreover, ID-1 plays various roles such as markers for progression, metastasis and prognosis in prostate [20,21], breast [22,23], gastric [24,25], esophageal [26] and uterine cervical cancers [27].

In a previous study, expression of ID-1 was shown as an independent prognostic factor in ovarian cancer with long-time follow-up. Overexpression of ID-1 is associated with more aggressive behavior of tumor cells in ovarian cancer [28]. However, no study has investigated the molecular function of ID-induced tumor progression in ovarian cancer. This prompted us to study the expression manner of ID proteins in ovarian cancers against clinical backgrounds with angiogenic potential in the tumors.

## Methods

### Patients and tissues

Prior informed consent for the following studies was obtained from all patients and approval was given by the Research Committee for Human Subjects, Gifu University School of Medicine. Sixty patients ranging from 34 to 83 years of age with ovarian cancers [stage I, 18 cases; stage II, 13 cases; and stage III, 15 cases; stage IV, 14 cases; 23 cases of serous papillary cystadenocarcinoma (SPCY), 8 cases of serous cystadenocarcinoma (SCY), 10 cases of mucinous cystadenocarcinoma (MCY), 8 cases of clear cell adenocarcinoma (C) and 11 cases of endometrioid adenocarcinoma (E)] underwent surgery at the Department of Obstetrics and Gynecology, Gifu University School of Medicine, between December 1997 and January 2004. Patient prognosis was analyzed in relation to a 36-month survival rate. None of the patients had received any pre-operative therapy before the ovarian cancer tissue was taken in surgery. A part of each tissue of ovarian cancers was snap-frozen in liquid nitrogen and stored at -80°C to determine ID-1, ID-2 and ID-3 mRNA levels and those for immunohistochemistry were fixed with 10% formalin and embedded in paraffin wax. The clinical stage of ovarian cancers was determined by International Federation of Obstetrics and Gynecology (FIGO) classification [29].

### Immunohistochemistry

Sections (4 μm) of formalin-fixed paraffin-embedded tissue samples from ovarian cancers were cut with a microtome and dried overnight at 37°C on a silanized-slide (Dako, Carpinteria, CA, USA). The protocol of universal Dako-Labelled Streptavidin-Biotin kit (Dako, Carpinteria, CA, USA) was followed for each sample. Samples were deparaffinized in xylene at room temperature for 30 min, rehydrated with graded ethanol and washed in phosphate-buffered saline (PBS). The samples were then placed in 10 mM citrate buffer (pH 6.0) and boiled in a microwave for 10 min for epitope retrieval. Endogenous peroxidase activity was quenched by incubating tissue sections in 3% H_2_O_2 _for 10 min. The primary antibodies, rabbit antihuman ID-1 (SC-734, Santa Cruz Biotechnology Inc., Santa Cruz, CA, USA), mouse CD34 (Dako, Glostrup, Denmark) and rabbit anti-factor VIII-related antigen (Zymed, San Francisco, CA, USA) were used overnight at 4°C at dilutions of 1:50, 1:40 and 1:2, respectively. The slides were washed and biotinylated secondary antibody (Dako, Carpinteria, CA, USA) was applied for 30 min after rinsing in PBS, after which streptavidin-conjugated horseradish peroxidase (Dako, Carpinteria, CA, USA) was added for 30 min. Slides were then washed and treated with the chromogen 3,3'-diaminobenzidine (Dako, Carpinteria, CA, USA) for 5 min, then rinsed in PBS, and counterstained with Mayer's haematoxylin, dehydrated in graded ethanols, cleared in xylene and cover-slipped with a mounting medium, Entellan New (Merck, Darmstadt, Germany). For confirmation of the specificity for ID-1 antigen, we also used another ID-1 (SC-488) rabbit polyclonal antibody (Santa Cruz Biotechnology Inc., Santa Cruz, CA, USA) and we have observed the exact identified intensity and localization of staining for ID-1 expression in tumor cells as ID-1 (SC-734) antibody. For the negative controls, the primary antibodies of ID-1, CD34 and factor VIII-related antigen were omitted and the corresponding preimmune animal serums (rabbit, mouse and rabbit, respectively) (Dako, Carpinteria, CA, USA) were used instead.

### Assessment of histochemical score (histoscore)

All sections of immunohistochemical staining for ID-1 were evaluated in a semiquantitative fashion according to the method described by McCarty et al. [30], which considers both the intensity and the percentage of cells stained in each of five intensity categories. Intensities were classified as 0 (no staining), 1 (weak staining), 2 (distinct staining), 3 (strong staining) and 4 (very strong staining). For each stained section, a value-designated histoscore was obtained by application of the following algorithm: histoscore = Σ(*i*+1) × *Pi*, where *i and Pi *represent intensity and percentage of cells that stain at each intensity, respectively, and corresponding histoscores were calculated separately. Results were assigned to four groups according to their overall scores: weak, <160; distinct, 161<, >220; strong, 221<, >280; very strong, 280<.

### Assessment of microvessel density (MVD)

The MVD was assessed with microvessel counts (MVCs) in sequential tissue sections stained with mouse CD34 and rabbit factor VIII-related antigen antibodies. Blood vessels with a clearly defined lumen or a well defined linear vessel shape, but not single endothelial cells, were taken into account for microvessel counting [31]. Fives areas of highest vascular density were chosen and microvessel counting was performed at ×200 magnification by two investigators. The MVCs were determined as the mean of the vessel counts obtained from these fields [32].

### Preparation of standard template for real-time reverse transcription-polymerase chain reaction (RT-PCR)

Internal standard template for real-time PCR was produced by PCR amplification using the primers of ID-1 gene, 418-782 in the cDNA (ID-1-TS: 5'-TTGGAGCTGAACTCGGAA-3' and ID-1-TAS: 5'-TCTCTGGTGACTAGTAGGT-3'); ID-2 gene, 907-1253 in the cDNA (ID-2-TS: 5'-CTAAGCAGACTTTGCCTTT-3' and ID-2-TAS: 5'-CTGAAATAAAGCAGGCAATC-3'); ID-3 gene, 686-1009 in the cDNA (ID-3-TS: 5'-GAACTTGTCATCTCCAACGA-3' and ID-3-TAS: 5'-CACGCTCTGAAAAGACCT-3'). The DNA template was purified using a GeneClean II kit (Qbiogene, Irvine, CA, USA). The copy numbers of the standard template were determined to quantitate ID-1, ID-2 and ID-3 mRNA level in samples for real-time RT-PCR.

### Real-time RT-PCR to amplify ID-1, ID-2 and ID-3 mRNAs

Total RNA was extracted with the acid guanidinium thiocyanate-phenol-chloroform method [33]. The total RNA (3 μg) was reverse transcribed using Moloney murine leukemia virus reverse transcriptase (MMLV-RT, 200 U/μl, Invitrogen, Carlsbad, CA, USA) and the following reagents: 250 mM Tris-HCl, pH 8.3, 375 mM KCl, 15 mM MgCl_2_, 0.1 M dithiothreitol, 10 mM deoxynucleotide [deoxyadenosine, deoxythymidine, deoxyguanosine and deoxycystidine] tri-phosphates (dNTPs) mixture and random hexamers (Invitrogen) at 37°C for 1 h. The reaction mixture was heated for 5 min at 94°C to inactivate MMLV-RTase.

Real-time PCR reaction was performed with a Takara Premix Ex Taq (Perfect Real Time) R-PCR kit (Takara, Otsu, Japan), using a smart cycler system (Cepheid, Sunnyvale, CA, USA). The reaction solution (25 μl) contained Takara Premix Ex Taq (2×), SYBR Green I (1:1000 dilution; CambrexBio Science, Rockland Inc., Rockland, ME, USA) and 20 μM of the primers of ID-1 gene, 545-675 in the cDNA (ID-1-S: 5'-ACGATCGCATCTTGTGTC-3' and ID-1-AS: 5'-CTTGTTCTCCCTCAGATCC-3'); ID-2 gene, 907-1026 in the cDNA (ID-2-S: 5'-CTAAGCAGACTTTGCCTTT-3' and ID-2-AS: 5'-CATTCAGTAGGCTTGTGTC-3'); ID-3 gene, 709-873 in the cDNA (ID-3-S: 5'-AAGGAGCTTTTGCCACTGA-3' and ID-3-AS:5'-CCAGGAAGGGATTTGGTGAA-3') with the transcribed total RNA from the tissue and a serially diluted standard template. The real-time PCR reactions were initially denatured by heating at 95°C for 30 s, followed by 40 cycles consisting of denaturation at 94°C for 10 s, annealing at 55°C for 5 s and extension at 72°C for 20 s. A strong linear relationship between the threshold cycle and the log concentration of the starting DNA copy number was always shown (correlation coefficient > 0.99). Quantitative analysis was performed to determine the copy number of each sample.

### Statistical analysis

ID-1, ID-2 and ID-3 mRNA levels were determined from three parts taken from each tumor, and each sample was analyzed in triplicate. Statistical analysis was performed using MedCalc Software version 9.2.0.1. ID-1 histoscores and mRNA levels were compared using the Mann-Whitney test and the Kruskal-Wallis test as appropriate. The 36-month survival rate was calculated according to the Kaplan-Meier method. The log-rank test and the Cox proportional hazards model were used for univariate and multivariate analyses of overall survival, respectively. The correlations between ID-1 histoscores and mRNA levels with MVCs were performed with Spearman's coefficient of correlation just for descriptive analysis. Differences were considered significant when *P *was less than 0.05.

## Results

**Figure 1:**
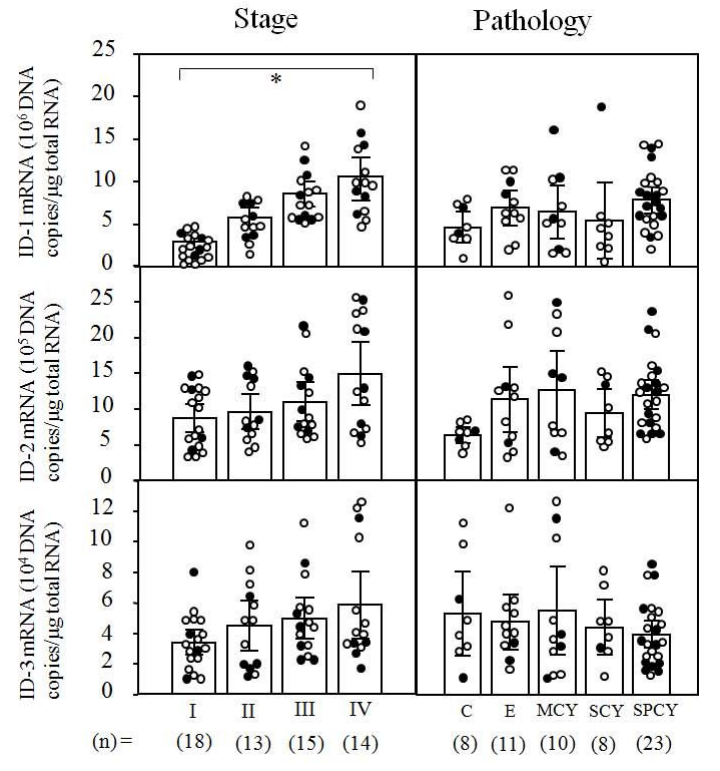
**ID-1, ID-2 and ID-3 mRNA levels in ovarian cancers classified according to clinical stage and histopathological type**. Clinical staging of ovarian cancers was done according to FIGO. Each level is the mean ± SD of nine determinations. C, clear cell carcinoma; E, endometrioid adenocarcinoma; MCY, mucinous cystadenocarcinoma; SCY, serous cystadenocarcinoma, SPCY, serous papillary cystadenocarcinoma. Alive and deceased cases are numbered in open circles and closed circles, respectively. * p < 0.001.

ID-1 mRNA levels significantly increased with increasing clinical stages (p < 0.001) of ovarian cancers, regardless of histopathological type (Figure 1). However, there was no significant difference in ID-2 or ID-3 mRNA levels according to clinical stage or histopathological type in ovarian cancers, as shown in Figure 1. These results prompted us to concentrate our investigation on ID-1 in ovarian cancers.

**Figure 2:**
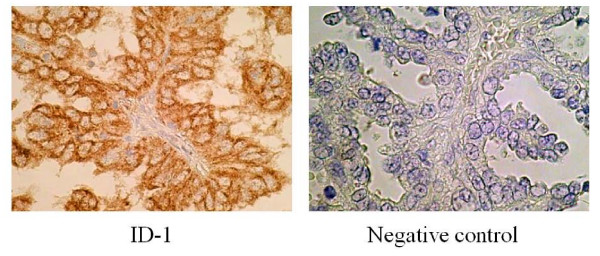
**Immunohistochemical staining for ID-1 with negative control in ovarian cancers**. A representing case of clear cell carcinoma of the ovary. Rabbit anti-human ID-1 (SC-734, Santa Cruz Biotechnology Inc., Santa Cruz, CA, USA) was used at a dilution of 1:50 as the primary antibody. (original magnification ×400).

Although ID-1 expression in stroma cells was negative, ID-1 staining was diffusely located in the cancer cells (Figure 2, a representative case of clear cell carcinoma of ovary). Because ID-1 is not a transcription factor *per se*, it lacks the nuclear localization signal found on many basic HLH proteins but gives a cytoplasmic signal instead [34,35]. ID-1 diffuse cytoplasmic staining was seen from moderate to strong intensity in most cases whereas nuclear staining was observed only occasionally (Figure 2).

**Figure 3:**
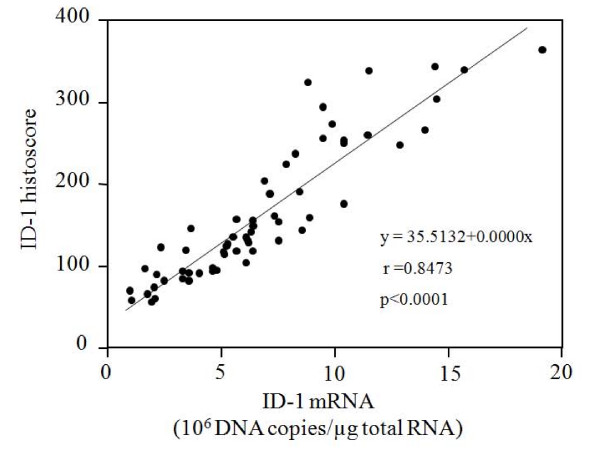
**Correlation between ID-1 histoscores in cancer cells and mRNA (10^6 ^DNA copies/μg total RNA) levels in ovarian cancers**. ID-1 histoscores and mRNA levels were determined by immunohistochemistry and real-time RT-PCR, respectively. Each level is the mean ± SD of nine determinations.

**Figure 4:**
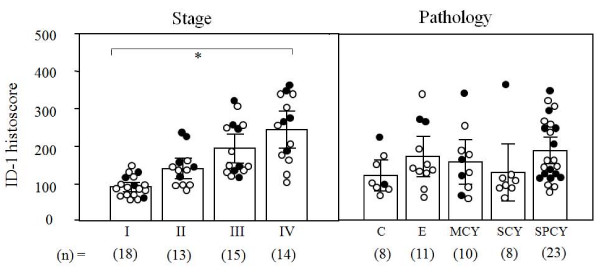
**ID-1 histoscores in ovarian cancers classified according to clinical stage and histopathological type**. Clinical staging of ovarian cancers was done according to FIGO. Each level is the mean ± SD of nine determinations. C, clear cell carcinoma; E, endometrioid adenocarcinoma; MCY, mucinous cystadenocarcinoma; SCY, serous cystadenocarcinoma, SPCY, serous papillary cystadenocarcinoma. Alive and deceased cases are numbered in open circles and closed circles, respectively. * p < 0.001.

ID-1 histoscore in cancer cells significantly (p < 0.001) correlated with the corresponding mRNA levels in each tissue, as shown in Figure 3. Although there was no significant difference in ID-1 histoscores in cancer cells according to histopathological type, ID-1 histoscores significantly (p < 0.001) increased with increased clinical stages of ovarian cancers (Figure 4), as did ID-1 mRNA.

**Figure 5:**
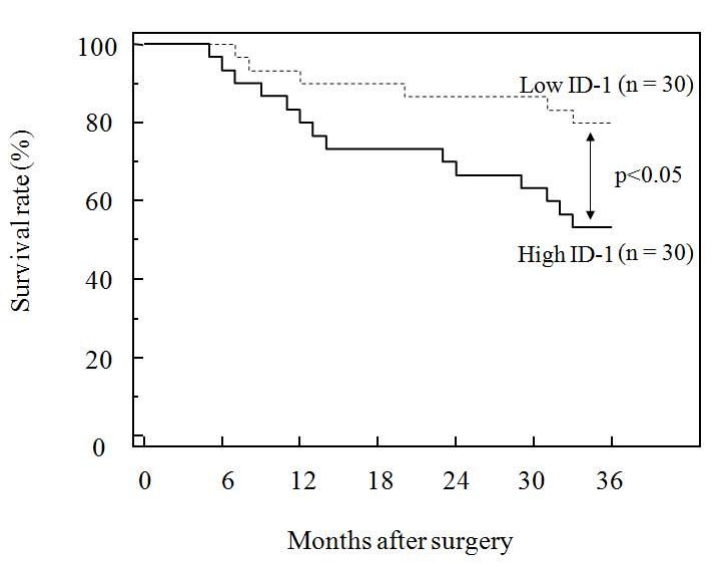
**Survival rate after surgery for ovarian cancers**. Patient prognosis was analyzed with a 36-month survival rate. High ID-1, cases with high histoscores and mRNA levels (> 160 histoscore and > 6.2 × 10^6^copies/μg total RNA, respectively). Low ID-1, cases with low histoscores and mRNA levels (< 160 histoscore and < 6.2 × 10^6 ^copies/μg total RNA, respectively).

Furthermore, the 60 patients who underwent surgery were divided into two equal groups based on ID-1 histoscores and mRNA levels, with the midpoint being a histoscore of 160 and mRNA of 6.2 × 10^6 ^copies/μg total RNA, respectively. The two groups, determined independently by the ID-1 histoscores and mRNA levels, consisted of exactly the same patients. The prognosis of the 30 patients with high ID-1 (> 160 histoscore and > 6.2 × 10^6 ^copies/μg total RNA) in ovarian cancers was poor (53%), whereas the 36-month survival rate of the other 30 patients with low ID-1 (< 160 histoscore and < 6.2 × 10^6 ^copies/μg total RNA) was higher (80%), as shown in Figure 5.

**Figure 6:**
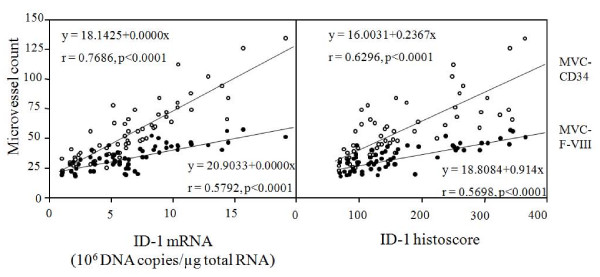
**Correlation of ID-1 histoscores in cancer cells and mRNA levels in ovarian cancers with microvessel counts (MVCs)**. White circles, (MVC-CD34) by immunohistochemical staining for CD34; black circles, (MVC-FV-III) by immunohistochemical staining for factor VIII-related antigen. Each level is the mean ± SD of nine determinations.

ID-1 expression was negative in endothelial cells, although CD34 and Factor VIII-related antigen expressions were strong. ID-1 histoscores significantly correlated with MVC-CD34 (MVCs determined by immunohistochemistry for CD34; r = 0.6296, p < 0.0001) and MVC-F-VIII (MVCs determined by immunohistochemistry for factor VIII-related antigen; r = 0.5698, p < 0.0001), and ID-1 mRNA levels also correlated with MVC-CD34 (r = 0.7686, p < 0.0001) and MVC-F-VIII (r = 0.5792, p < 0.0001), as shown in Figure 6.

## Discussion and conclusion

In the present study, ID-1 expression increased with tumor advancement of ovarian cancers and patients with high ID-1 expression had a lower survival rate compared to patients with low ID-1 expression. Previously, no correlation between expression of ID proteins and angiogenesis, assessed by MVD was observed in ovarian cancers [28].

Ectopic expression of ID-1 in a prostate cancer cell line that express low levels of both ID-1 and VEGF protein resulted increase in VEGF secretion by the cells, which is associated with increased VEGF gene transcription, after ID-1 gene transfection [36]. ID-1 promotes tumor angiogenesis through VEGF gene transcription and protein expression. In addition, VEGF is a downstream target of the ID-1 protein [36]. ID-1 promotes tumor angiogenesis through induction of the VEGF gene transcription and protein expression [37-39], leading to proliferation and endothelial tube formation of the vascular endothelial cells. ID-1 induces activity of HIF-1α in human endothelial and breast cancer cells [40,41]. Furthermore, ID-1 enhanced nuclear translocation and the transcriptional activity of HIF-1α [42]. In a previous study, increased HIF-1α levels were observed with tumor advancement and poor patient prognosis in uterine cervical cancers [43].

When tumor xonografts were implanted into ID-1/ID-3 mice, decreased tumor growth as well as loss of metastasis were observed, which were associated with impaired neovascularization of tumor [19]. In ID-1/- mice, up-regulation of a potent angiogenic inhibitor thrombospondin-1 in null embryonic fibroblasts was observed [44]. This showed ID-1 alone was sufficient to impair tumor angiogenesis. In the present study, ID-1 expression correlated with microvessel counts indicating that ID-1 overexpression contributed to tumor angiogenesis in ovarian cancer. Therefore, ID-1 is a candidate for angiogenic mediator as the clinical relevance of angiogenesis assessed by MVC.

In conclusion, this study demonstrates the functional role of ID-1 overexpression in tumor advancement via angiogenesis and ID-1 can be a useful prognostic indicator in ovarian cancers. In addition, ID-1 protein might be an important new target molecule for anti-angiogenic drug design in cancer treatment.

## Competing interests

The authors declare that they have no competing interests.

## Authors' contributions

MKM has made substantial contributions to conception and design, acquisition of data, analysis and interpretation of data. JF conceived of the study, participated in its design and in drafting the manuscript and revising it critically for important intellectual content. TT has given final approval of the version to be published. All authors read and approved the final manuscript.

## Pre-publication history

The pre-publication history for this paper can be accessed here:

http://www.biomedcentral.com/1471-2407/9/430/prepub
